# Supplementary material for: Multicomponent and multisensory communicative acts in orang-utans may serve different functions
Source: Commun Biol. 2021 Jul 27;4:917. doi: 10.1038/s42003-021-02429-y (PMC8316500; doi:10.1038/s42003-021-02429-y)
Supplement: Supplementary file 2 — Supplementary Tables and Figures [file 42003_2021_2429_MOESM2_ESM.pdf]

Supplementary Material

Multicomponent and multisensory communicative acts in orang-utans may serve different functions

Marlen Fröhlich, Natasha Bartolotta, Caroline Fryns, Colin Wagner, Laurene Momon, Marvin Jaffrezic, Tatang Mitra Setia, Maria van Noordwijk, Carel P. van Schaik

Additional information

Results

Use of specific articulators. We used GLMMs to test sources of variation (i.e. species, setting, kinship) in the use of communicative acts involving specific articulators. The full model was a better fit than the null model for the response variables manual, bodily, vocal and recipient-directed gaze acts (LRT for manual: $\chi^2_5 = 45.191$, $P < 0.001$; bodily: $\chi^2_5 = 74.46$, $P < 0.001$; gaze: $\chi^2_5 = 174.296$, $P < 0.001$, vocal acts: $\chi^2_5 = 15.166$, $P = 0.01$). More specifically, we found a significant interaction between species and setting for the use of bodily acts and gaze: bodily acts played a larger role in captive Sumatrans compared to the other species-setting classes (Tab. S1, Fig. S1), whereas recipient-directed gaze accompanied interactions significantly less often in captive compared to wild orang-utans (Tab. S1, Fig. S2). We also found a significant impact of kinship: manual acts were significantly more common in interactions among mother-infant dyads (Tab. S1) and other maternal kin, whereas communicative acts among mother-infant dyads were less likely to involve recipient-directed gaze (Tab. S1, Fig. S2) than those among other interaction dyads. Moreover, vocal acts were more commonly used in interactions beyond the mother-offspring dyad. For effects of non-significant key predictors and those of control variables see Table S1.

Use of specific modalities. Next**,** we used GLMMs to test sources of variation (i.e. species, setting, kinship) in the use of communicative acts involving specific modalities. The full model was a better fit than the null model for visual and tactile acts only (LRT for visual acts: $\chi^2_5 = 69.229$, $P < 0.001$; tactile acts: $\chi^2_5 = 106.53$, $P < 0.001$; $N = 7587$). More specifically, communicative acts were more likely to contain visual acts in the wild, in the Sumatran orang-utans, and in interaction dyads other than mother-offspring or maternal kin (Tab. S1). For tactile acts, there was a significant interaction between species and setting: wild Bornean orang-utans employed more tactile acts than any other species-setting combination (Tab. S1, Fig. S3). Tactile acts were significantly more common in mother-infant interactions compared to other dyads (Tab. S1, Fig. S3). For effects of the control variables see Table S5.

Tables

Tab. S1 Effects of research setting, orang-utan species, kinship and control predictors on the use of different articulators (a-d) and sensory modalities (e-f), derived using GLMMs with a binomial error structure (N = 7587). Significant effects (P < 0.05) are depicted in italics.

**(a) Manual**	Estimate	SE	χ^2_1	P
Intercept	0.669	0.251	—	—
Setting [wild]	0.42	0.173	5.710	0.017
Species [Sumatran]	-0.233	0.184	1.581	0.209
Age group [young im.]	-0.395	0.2	3.825	0.050
Age group [older im.]	-0.269	0.213	1.560	0.212
Sex [male]	0.14	0.17	0.668	0.414
Kinship [mother]	*1.003*	*0.207*	*22.414*	*<0.001*
Kinship [mat. kin]	*0.556*	*0.192*	*8.227*	*0.004*
Context [share]	*-1.102*	*0.143*	*49.474*	*<0.001*
Context [play]	-0.186	0.128	2.027	0.155
**(b) Bodily**				
Intercept	-2.168	0.282	—	—
Setting [wild]	-0.691	0.271	—	—
Species [Sumatran]	0.584	0.246	—	—
Age group [young im.]	0.304	0.183	2.772	0.096
Age group [older im.]	0.172	0.199	0.747	0.388
Sex [male]	0.11	0.153	0.513	0.474
Kinship [mother]	-0.09	0.192	0.221	0.638
Kinship [mat. kin]	0.017	0.179	0.009	0.922
Context [share]	*1.645*	*0.158*	*73.177*	*<0.001*
Context [play]	*0.661*	*0.132*	*23.346*	*<0.001*
Setting x species	*-0.832*	*0.335*	*5.980*	*0.014*
**(c) Gaze**				
Intercept	-2.114	0.308	—	—
Setting [wild]	4.022	0.307	—	—
Species [Sumatran]	2.781	0.278	—	—
Age group [young im.]	-0.019	0.189	0.011	0.918
Age group [older im.]	0.307	0.208	2.181	0.140
Sex [male]	0.023	0.169	0.018	0.892
Kinship [mother]	*-0.866*	*0.202*	*17.886*	*<0.001*
Kinship [mat. kin]	-0.093	0.193	0.236	0.627
Context [share]	*-1.539*	*0.167*	*65.507*	*<0.001*
Context [play]	*0.478*	*0.124*	*14.903*	*<0.001*
Setting x species	*-2.589*	*0.365*	*45.187*	*<0.001*
**(d) Vocal**				
Intercept	-9.041	2.075	—	—
Setting [wild]	0.352	1.483	0.055	0.815
Species [Sumatran]	-2.505	1.583	2.223	0.136
Age group [young im.]	*3.849*	*1.62*	*5.394*	*0.020*
Age group [older im.]	1.999	1.741	1.203	0.273
Sex [male]	*-3.083*	*1.416*	*4.874*	*0.027*
Kinship [mother]	*-6.441*	*2.041*	*11.130*	*0.001*
Kinship [mat. kin]	-2.494	1.829	1.960	0.161

Context [share]	*-3.964*	*1.912*	*10.439*	*0.001*
Context [play]	*-5.846*	*1.535*	*22.286*	*<0.001*
**(e) Visual**				
Intercept	-0.786	0.189	—	—
Setting [wild]	*0.763*	*0.126*	*33.396*	*<0.001*
Species [Sumatran]	*0.583*	*0.13*	*18.065*	*<0.001*
Age group [young im.]	0.179	0.139	1.599	0.206
Age group [older im.]	0.152	0.155	0.939	0.332
Sex [male]	-0.176	0.117	2.241	0.134
Kinship [mother]	*-0.794*	*0.147*	*28.780*	*<0.001*
Kinship [mat. kin]	*-0.636*	*0.141*	*19.755*	*<0.001*
Context [share]	*1.933*	*0.14*	*134.821*	*<0.001*
Context [play]	*0.23*	*0.095*	*5.725*	*0.017*
**(f) Tactile**				
Intercept	0.546	0.296	—	—
Setting [wild]	1.861	0.301	—	—
Species [Sumatran]	0.18	0.26	—	—
Age group [young im.]	-0.008	0.193	0.002	0.969
Age group [older im.]	0.027	0.21	0.017	0.897
Sex [male]	*0.366*	*0.163*	*4.925*	*0.026*
Kinship [mother]	*1.002*	*0.206*	*22.243*	*<0.001*
Kinship [mat. kin]	0.368	0.188	3.834	0.050
Context [share]	*-0.803*	*0.175*	*20.652*	*<0.001*
Context [play]	0.067	0.125	0.287	0.592
Setting x species	*-2.53*	*0.367*	*42.966*	*<0.001*

Tab. S2 Effects of research setting, orang-utan species, kinship and control predictors on the composition of communicative acts (a: multicomponent [MC] use of unisensory [US] acts, b: multisensory [MS] use of unicomponent [UC] acts, c: MC use of MS acts, d: MS use of MC acts) derived using GLMMs with a binomial error structure (a: N = 5239, b: N = 4954, c: N = 2348, d: N = 2633,). Significant effects (P < 0.05) are depicted in italics.

**(a) MC-US vs UC-US**	Estimate	SE	χ^2_1	*P*
Intercept	-2.537	0.326	—	—
Setting [wild]	2.359	0.314	—	—
Species [Sumatran]	1.99	0.291	—	—
Age group [young im.]	-0.287	0.183	2.408	0.121
Age group [older im.]	-0.11	0.193	0.324	0.569
Sex [male]	-0.226	0.154	2.121	0.145
Kinship [mother]	*-1.071*	*0.193*	*28.144*	*<0.001*
Kinship [mat. kin]	0.035	0.18	0.038	0.845
Context [share]	*-0.527*	*0.168*	*11.239*	*0.001*
Context [play]	0.109	0.139	0.616	0.433
Setting x species	*-1.08*	*0.356*	*9.456*	*0.002*
**(b) MS-UC vs US-UC**				
Intercept	-4.584	0.388	—	—
Setting [wild]	3.129	0.336	—	—
Species [Sumatran]	1.342	0.334	—	—
Age group [young im.]	*0.598*	*0.181*	*10.132*	*0.001*
Age group [older im.]	0.306	0.223	1.900	0.168
Sex [male]	0.031	0.154	0.042	0.838
Kinship [mother]	-0.046	0.2	0.052	0.819
Kinship [mat. kin]	-0.13	0.208	0.394	0.530
Context [share]	*1.548*	*0.184*	*45.124*	*<0.001*
Context [play]	0.18	0.143	1.602	0.206
Setting x species	*-1.306*	*0.391*	*12.041*	*0.001*
**(c) MC-MS vs UC-MS**				
Intercept	-1.429	0.836	—	—
Setting [wild]	0.665	0.8	—	—
Species [Sumatran]	2.458	0.815	—	—
Age group [young im.]	-0.387	0.304	1.620	0.203
Age group [older im.]	-0.039	0.374	0.011	0.918
Sex [male]	0.012	0.257	0.002	0.964
Kinship [mother]	*-0.627*	*0.314*	*3.987*	*0.046*
Kinship [mat. kin]	-0.589	0.329	3.210	0.073
Context [share]	*-2.301*	*0.522*	*29.094*	*<0.001*
Context [play]	*0.703*	*0.239*	*9.309*	*0.002*
Setting x species	*-2.071*	*0.866*	*6.049*	*0.014*
**(d) MS-MC vs US-MC**				
Intercept	-2.988	0.701	—	—
Setting [wild]	1.461	0.68	—	—
Species [Sumatran]	1.225	0.669	—	—
Age group [young im.]	0.212	0.281	0.565	0.452
Age group [older im.]	0.26	0.309	0.705	0.401
Sex [male]	0.212	0.224	0.895	0.344
Kinship [mother]	*0.558*	*0.279*	*3.933*	*0.047*

Kinship [mat. kin]	*-0.914*	*0.263*	*11.526*	*0.001*
Context [share]	0.333	0.396	0.627	0.429
Context [play]	*0.447*	*0.201*	*5.101*	*0.024*
Setting x species	*-1.916*	*0.726*	*7.664*	*0.006*

Tab. S3 Effects of multisensory (MS; a: visual plus, b: tactile plus) or multicomponent use (MC; c: recipient-directed gaze plus) of communicative acts and control predictors on the probability of receiving an apparently satisfactory outcome (ASO), derived using GLMMs with a binomial error structure (a: *N* = 2301, b: *N* = 3743, c: *N* = 4513). Significant effects (P < 0.05) are depicted in italics.

**(a) ASO ~ Visual plus**	Estimate	SE	χ^2_1	*P*
Intercept	0.724	0.563	—	—
MS use [visual plus]	*0.89*	*0.211*	*14.458*	*<0.001*
Setting [wild]	-0.127	0.466	0.074	0.785
Species [Sumatran]	*-1.372*	*0.496*	*4.395*	*0.036*
Age group [young im.]	*-0.842*	*0.31*	*7.510*	*0.006*
Age group [older im.]	-0.531	0.337	2.538	0.111
Sex [male]	0.483	0.257	3.528	0.060
Kinship [mother]	*0.918*	*0.308*	*9.066*	*0.003*
Kinship [mat. kin]	0.463	0.319	2.122	0.145
Context [share]	*-0.949*	*0.277*	*10.523*	*0.001*
Context [play]	0.242	0.287	0.721	0.396
**(b) ASO ~ Tactile plus**				
Intercept	-0.462	0.463	—	—
MS use [tactile plus]	*0.549*	*0.161*	*9.692*	*0.002*
Setting [wild]	0.616	0.413	2.397	0.122
Species [Sumatran]	-0.454	0.424	1.330	0.249
Age group [young im.]	-0.036	0.285	0.036	0.850
Age group [older im.]	-0.148	0.312	0.007	0.935
Sex [male]	0.341	0.269	1.217	0.270
Kinship [mother]	*0.778*	*0.275*	*10.661*	*0.001*
Kinship [mat. kin]	0.295	0.272	0.156	0.693
Context [share]	-0.243	0.192	1.361	0.243
Context [play]	0.377	0.229	1.900	0.168
**(c) ASO ~ Gaze plus**				
Intercept	0.535	0.271	—	—
MC use [gaze plus]	*0.492*	*0.117*	*15.810*	*<0.001*
Setting [wild]	0.139	0.181	0.587	0.443
Species [Sumatran]	*-0.995*	*0.191*	*25.631*	*<0.001*
Age group [dependent]	-0.289	0.206	1.960	0.161
Age group [immature]	0.056	0.226	0.061	0.806
Sex [male]	0.12	0.181	0.447	0.504
Kinship [mother]	*0.632*	*0.22*	*8.384*	*0.004*
Kinship [mat. kin]	0.037	0.206	0.033	0.856
Context [share]	*-1.149*	*0.192*	*36.053*	*<0.001*
Context [play]	0.233	0.151	2.426	0.119

Tab. S4 Effects of multicomponent (MC) use of communicative acts (a: bodily plus, b: recipient-directed gaze plus) and control predictors on the probability of matching the dominant outcome (DOM) of a communicative act, derived using GLMMs with a binomial error structure (a: N = 1429, b: N = 3869). Significant effects (P < 0.05) are depicted in italics.

**(a) DOM ~ Bodily plus**	Estimate	SE	χ^2_1	P
Intercept	-1.82	0.727	—	—
MC use [bodily plus]	*-0.907*	*0.414*	*4.690*	*0.030*
Setting [wild]	-0.483	0.541	0.785	0.376
Species [Sumatran]	-0.59	0.564	1.115	0.291
Age group [young im.]	0.762	0.668	1.319	0.251
Age group [older im.]	0.679	0.71	0.910	0.340
Sex [male]	1.202	0.619	3.821	0.051
Kinship [mother]	0.483	0.365	1.754	0.185
Kinship [mat. kin]	0.582	0.387	2.261	0.133
Context [share]	*5.797*	*0.518*	*162.881*	*<0.001*
Context [play]	*3.697*	*0.37*	*131.890*	*<0.001*
**(b) DOM ~ Gaze plus**				
Intercept	-0.621	0.537	—	—
MC use [gaze plus]	*-0.668*	*0.257*	*6.560*	*0.010*
Setting [wild]	-0.569	0.338	2.750	0.097
Species [Sumatran]	-0.311	0.337	0.840	0.360
Age group [young im.]	-0.221	0.366	0.360	0.547
Age group [older im.]	-0.005	0.401	0.000	0.990
Sex [male]	0.653	0.335	3.740	0.053
Kinship [mother]	0.245	0.223	1.210	0.272
Kinship [mat. kin]	*1.01*	*0.24*	*17.660*	*<0.001*
Context [share]	0.436	0.244	3.130	0.077
Context [play]	*5.049*	*0.194*	*1204.620*	*<0.001*

Tab. S5 Information on study subjects (i.e. signallers and recipients) and sample size. Individuals with zero communicative acts were only recipients, those marked with an asterisk (*) never acted as recipients (Bor = Bornean orang-utan, Sum = Sumatran orang-utan, Ad = adult, Oi = older immature, Yi = young immature).

No subject	Setting	Species	Group	ID	Age group	Sex	N Comm. acts
1	captive	Bor	Apenheul	WAT	Ad	F	67
2	captive	Bor	Apenheul	KAW	Oi	M	59
3	captive	Bor	Apenheul	BAJ	Yi	M	65
4	captive	Bor	Cologne	CAJ	Ad	F	42
5	captive	Bor	Cologne	COR	Ad	F	31
6	captive	Bor	Cologne	CIT	Oi	F	60
7	captive	Bor	Cologne	BUD	Oi	M	25
8	captive	Bor	Cologne	CIR	Yi	F	86
9	captive	Bor	Munster	MAN	Ad	F	34
10	captive	Bor	Munster	SAR	Ad	F	17
11	captive	Bor	Munster	NIA	Yi	F	12
12	captive	Bor	Munster	MIY	Yi	M	79
13	captive	Sum	Munich	JAH	Ad	F	58
14	captive	Sum	Munich	MAT	Ad	F	70
15	captive	Sum	Munich	SIT	Ad	F	129
16	captive	Sum	Munich	BRU	Ad	M	11
17	captive	Sum	Munich	ISO	Oi	F	132
18	captive	Sum	Munich	JOL	Oi	F	70
19	captive	Sum	Munich	RON	Yi	F	197
20	captive	Sum	Munich	QUE	Yi	M	287
21	captive	Sum	Munich	QUI	Yi	M	102
22	captive	Sum	Zurich	CAH	Ad	F	82
23	captive	Sum	Zurich	TIM	Ad	F	50
24	captive	Sum	Zurich	XIR	Ad	F	52
25	captive	Sum	Zurich	DJA	Ad	M	22
26	captive	Sum	Zurich	MIM	Oi	F	208
27	captive	Sum	Zurich	HAD	Oi	M	103
28	captive	Sum	Zurich	MAL	Oi	M	342
29	captive	Sum	Zurich	PAN	Yi	F	199
30	captive	Sum	Zurich	RIA	Yi	F	99
31	wild	Bor	Tuanan	CIA	Ad	F	45
32	wild	Bor	Tuanan	DES	Ad	F	144
33	wild	Bor	Tuanan	JUN	Ad	F	96
34	wild	Bor	Tuanan	KER	Ad	F	223
35	wild	Bor	Tuanan	KON	Ad	F	69
36	wild	Bor	Tuanan	MIL	Ad	F	119
37	wild	Bor	Tuanan	MIN	Ad	F	100
38	wild	Bor	Tuanan	TIN	Ad	F	59
39	wild	Bor	Tuanan	ZOL	Ad	F	1
40	wild	Bor	Tuanan	UNF	Ad	M	67
41	wild	Bor	Tuanan	MAW	Oi	F	46
42	wild	Bor	Tuanan	DAN	Oi	M	17
43	wild	Bor	Tuanan	TUK	Oi	M	125
44	wild	Bor	Tuanan	JAN	Yi	F	320

45	wild	Bor	Tuanan	MOB	Yi	F	390
46	wild	Bor	Tuanan	ZAK	Yi	F	16
47	wild	Bor	Tuanan	CAK	Yi	M	124
48	wild	Bor	Tuanan	DAR	Yi	M	330
49	wild	Bor	Tuanan	KEC	Yi	M	54
50	wild	Bor	Tuanan	KET	Yi	M	467
51	wild	Bor	Tuanan	MER	Yi	M	418
52	wild	Sum	Suaq	ALI	Ad	F	0
53	wild	Sum	Suaq	CIS	Ad	F	41
54	wild	Sum	Suaq	DOD	Ad	F	0
55	wild	Sum	Suaq	ELL	Ad	F	113
56	wild	Sum	Suaq	FRI	Ad	F	44
57	wild	Sum	Suaq	LIL	Ad	F	43
58	wild	Sum	Suaq	LIS	Ad	F	34
59	wild	Sum	Suaq	PIN	Ad	F	13
60	wild	Sum	Suaq	TIA	Ad	F	22
61	wild	Sum	Suaq	BEO	Ad	M	6
62	wild	Sum	Suaq	CRA	Ad	M	0
63	wild	Sum	Suaq	DRU	Ad	M	0
64	wild	Sum	Suaq	FLG	Ad	M	0
65	wild	Sum	Suaq	GUR	Ad	M	0
66	wild	Sum	Suaq	JAR	Ad	M	0
67	wild	Sum	Suaq	MAR	Ad	M	0
68	wild	Sum	Suaq	NIB	Ad	M	6
69	wild	Sum	Suaq	REE	Ad	M	0
70	wild	Sum	Suaq	ROB	Ad	M	0
71	wild	Sum	Suaq	UNF	Ad	M	6
72	wild	Sum	Suaq	CIN	Oi	F	225
73	wild	Sum	Suaq	TRI	Oi	F	0
74	wild	Sum	Suaq	YUL	Oi	F	8
75	wild	Sum	Suaq	FRA	Oi	M	371
76	wild	Sum	Suaq	KRO	Oi	M	45
77	wild	Sum	Suaq	LOI	Oi	M	164
78	wild	Sum	Suaq	EDE	Yi	F	391
79	wild	Sum	Suaq	AMO*	Yi	M	5
80	wild	Sum	Suaq	LUT	Yi	M	313
81	wild	Sum	Suaq	PEP	Yi	M	38
82	wild	Sum	Suaq	TOR	Yi	M	92

Tab. S6 Information on variables coded using BORIS

Variable and levels	Coding type	Description
**Presumed goal**	single-select	Apparent aim of the signaller (S) as determined by observer, based on the individuals involved and the signaller`s behaviour before and after the communicative act
Food/object share		Recipient (R) shares/hands over food item or object with signaller
Groom		R grooms S
Move away		R moves away from S
Play/affiliate		R plays or physically affiliates with
Sexual contact		R engages in sexual contact with S
Stop action		R stops a certain behaviour (e.g. begging) from S
Joint travel		R starts joint travel/co-locomotion with S
**Body part**	multi-select	Body part(s) used by the signaller to execute behaviour/gesture/etc.
Arm-R		Right arm
Arm-L		Left arm
Hand-R		Right hand
Hand-L		Left hand
Leg-R		Right leg
Leg-L		Left leg
Foot-R		Right foot
Foot-L		Left foot
Head		Head and eyes
Front		Front
Torso		Torso
Mouth/lips		Mouth/lips
Other		Other
NA		Na
**Modality**	multi-select	Sensory modality in which the signal is perceived by the recipient
Auditory		S's behaviour produces a sound and is perceived through hearing
Visual		S's behaviour is perceived via eye sight
Seismic		S's behaviour is perceived through substrate movement *(like vibration = indirect mechanic modality)*
Tactile		S's behaviour is perceived through body contact
**Distance**	single-select	Physical distance between S and R BEFORE signal/behaviour is executed (e.g. before R is touched)
Body contact		S and R are in body contact
Within arm's reach		No body contact, but S could (theoretically) touch R if he stretches out arm/leg
Same tree/substrate		S and R sit on/are attached to same substrate (e.g. Branch, liana, tree log)
Different tree/substrate		S and R sit on/are attached to different substrate (e.g. Branch, liana, tree log)
NA		Not clearly visible, unknown
**Objects**	single-select	Objects involved by signaller in behaviour/interaction
None		No object involved
Immobile		Immobile object involved, e.g. tree branch that is still attached to tree
Mobile		Mobile object involved, e.g. loose branch, stone, stick, leaf….
NA		Not clearly visible, unknown

**Gaze direction**	single-select	Visual orientation of signaller as determined by head/eye direction
At recipient		S’s head directed towards R
Mutual gaze		S’s head directed towards R and vice versa
At object		S’s head directed towards object (e.g. food item, toy) held/eaten by R
Alt between obj and rec		S alternates head direction between R and object
Other		S’s head directed neither towards R nor towards object held/eaten by R
NA		Not clearly visible, unknown
**Attentional state**	single-select	Visual orientation of recipient (R) during S's behaviour
In visual field		S is in the R’s visual field, i.e. The R can see the S without moving his or her head
90 degrees		S is in the periphery of the R’s visual field (ca. 90 degrees), i.e. R can see the S from the corner of his/her eye.
Rec turned away		S is not in the R’s visual field, that is the R would have to make a major turn with his/her head or body to see the S
NA		Not clearly visible, unknown
**Interaction outcome**	single-select	Response of recipient in reaction to S's behaviour
No reaction		No discernible response by R
ASO		"Apparently Satisfactory Outcome": R responds in a way that seems to satisfy S, in that no more communicative acts are produced
Responds w/ signal		R responds with a signal - "negotiation"
Visual attention		R’s head directed towards S, but no other response
Move away		R moves away from S
Agonistic		R reacts in an aggressive or agonistic way to S's behaviour
NA		Not clearly visible, unknown

Tab. S7 Overview of communicative acts for which dominant outcomes could be identified (FS = Share food/object, GR = Groom, JT = Co-locomote, PL = Play/affiliate, SX = Sexual contact, ST = stop action)

Comm. act	Type	No. subjects	Dominant outcome	Total
beg hand-hand	manual	36	FS	306
beg hand-mouth	manual	33	FS	223
beg mouth-hand	bodily	27	FS	173
beg mouth-mouth	bodily	30	FS	202
bite	bodily	47	PL	348
bite attempt	bodily	50	PL	237
dangle	bodily	33	PL	168
embrace	manual	23	PL	50
flapped lip	facial	6	ST	11
fling	manual	19	PL	72
grab/hold	manual	65	PL	1417
hand on	manual	47	PL, FS, GR	418
head-butt	bodily	10	PL	32
head-stand	bodily	3	PL	5
hit	manual	20	PL	91
kiss	bodily	37	PL	126
look	bodily	53	PL	348
look back	bodily	11	PL	27
loud scratch	manual	11	JT	34
peer	bodily	38	FS	361
play face	facial	26	PL	83
poke	manual	23	PL	126
pout face	facial	7	PL	16
present body part	manual	36	PL, GR	148
present object	manual	16	PL	27
pull	manual	61	PL, FS	548
push	manual	50	ST, PL	278
raise limb	manual	28	PL	52
reach	manual	53	PL, FS	236
rise up	bodily	7	PL	3
roll on back	bodily	7	PL	5
rub body	bodily	12	SX	87
shake object	manual	1	PL	5
somersault	bodily	6	PL	10
spin	bodily	3	PL	3
spit	bodily	1	PL	5
stroke	manual	11	PL	19
throw object	manual	7	PL	11
throw self	bodily	19	PL	72
touch	manual	66	PL	1045
Total		70		7428

Tab. S8 Distribution of coded interactions across settings, species and interaction dyads (mother-offspring: interactions among mothers and their dependent unweaned offspring; interactions among maternal kin other than mother-dependent offspring, other: all other interaction dyads).

	Captive		Wild		Total
Interaction dyad	Bornean	Sumatran	Bornean	Sumatran	
Mother-offspring	330	465	2208	1263	4266
Maternal kin	131	933	517	226	1807
Other	116	788	187	423	1514
**Total**	**577**	**2186**	**2912**	**1912**	**7587**

Tab. S9 Information on corresponding variable and level names in R script (ESM 3)

**Variable**	**Names of binary variables in R script**
Predictor variables	
Setting (captive, wild)	setting (captive, wild)
Species (Bornean, Sumatran)	species (Bor, Sum)
Age group: young immature	age.dep
Age group: older immature	age.imm
Sex: female, male	sex.code
Kinship: mother-offspring	kinship.mo
Kinship: maternal kin	kinship.mk
Presumed goal: share object/food	context_fs
Presumed goal: share object/food	context_pl
Constituent parts: visual plus, tactile plus, recipient-directed gaze plus, bodily plus, manual plus,	vis_mm, tac_mm, gaze_mp, bodily_mp, manual_mp
Response variables	
Articulators: bodily. manual, recipient-directed gaze, vocal, facial	artic_bod, artic_man, artic_gaze, , artic_voc, artic_fac
Modalities: visual, tactile. audible, seismic	modality_vis2, modality_tac, modality_aud, modality_seis
Multicomponent use of unisensorya cts	mc_us
Multisensory use of unicomponent acts	ms_uc
Multisensory use of multicomponent acts	MC_ms
Multicomponent use of multisensory acts	MS_mc
Effectiveness (ASO)	effect
Dominant outcome match (DOM)	match_tight

Figures

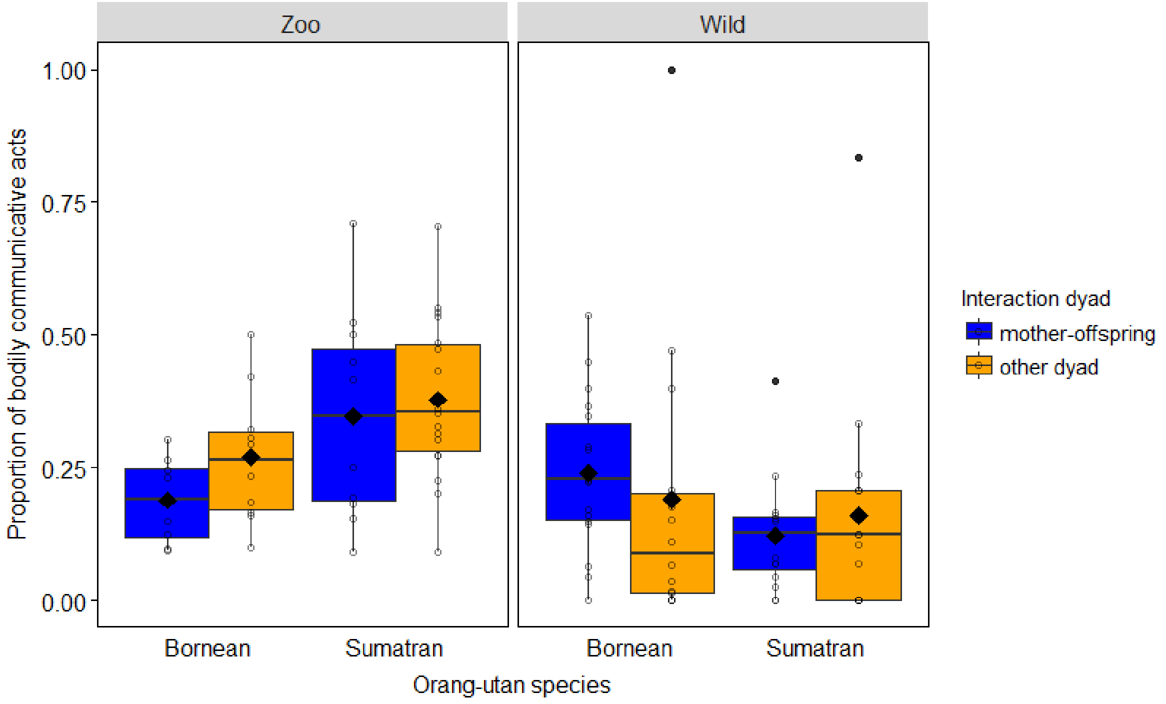

Fig. S1 Proportion of bodily acts as a function of research setting, species and interaction dyad. Indicated are individual means (circles), population means (filled diamonds), medians (horizontal lines), quartiles (boxes), percentiles (2.5% and 97.5%, vertical lines) and outliers (filled dots).

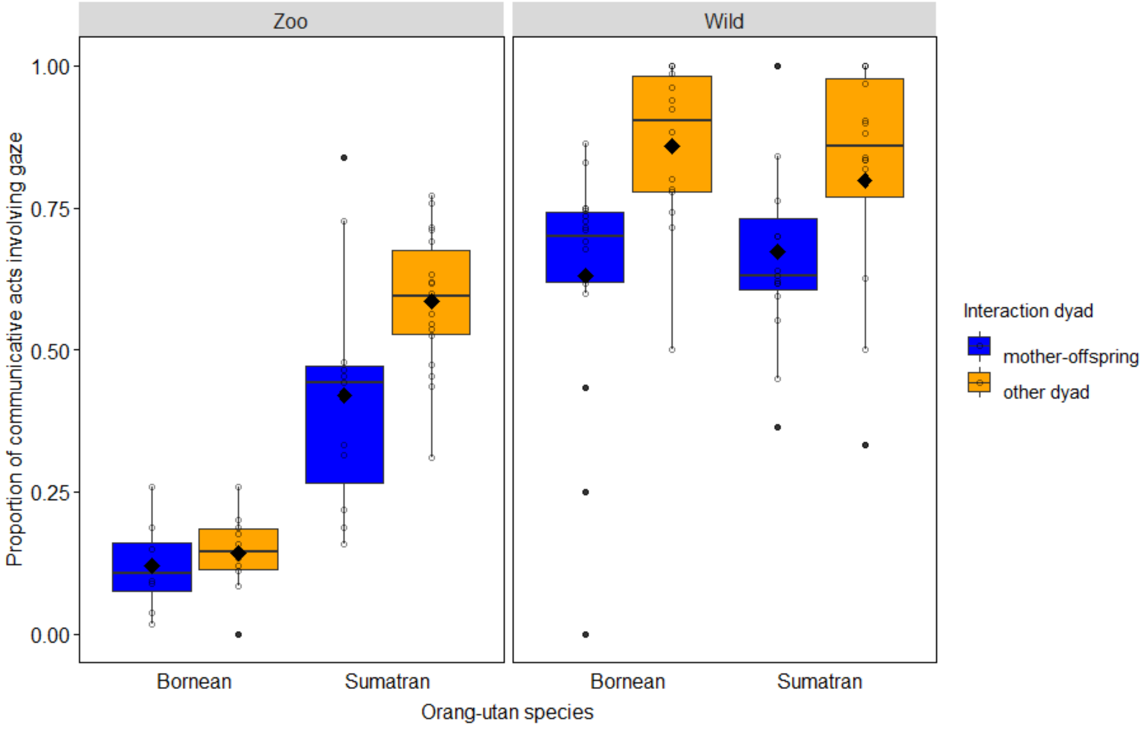

Fig. S2 Proportion of communicative acts involving recipient-directed gaze as a function of research setting, species and interaction dyad. Indicated are individual means (circles), population means (filled diamonds), medians (horizontal lines), quartiles (boxes), percentiles (2.5% and 97.5%, vertical lines) and outliers (filled dots).

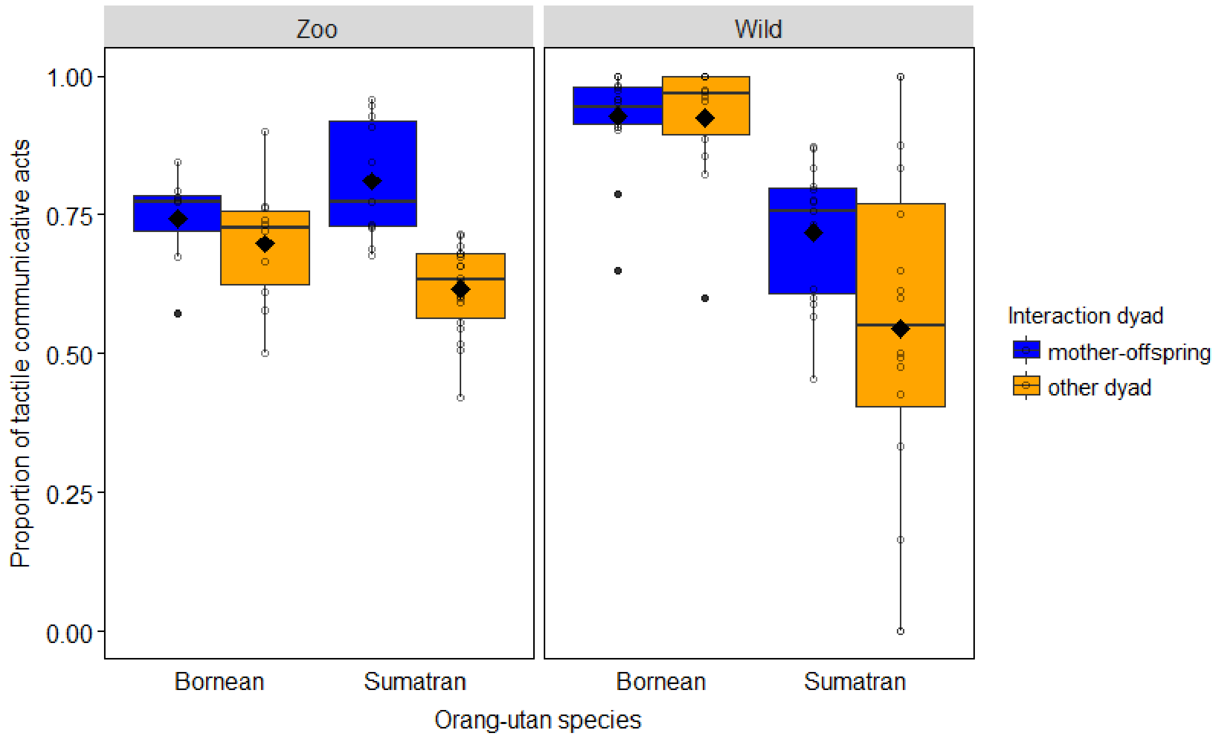

Fig. S3 Proportion of tactile communicative acts as a function of research setting, species and interaction dyad. Indicated are individual means (circles), population means (filled diamonds), medians (horizontal lines), quartiles (boxes), percentiles (2.5% and 97.5%, vertical lines) and outliers (filled dots).

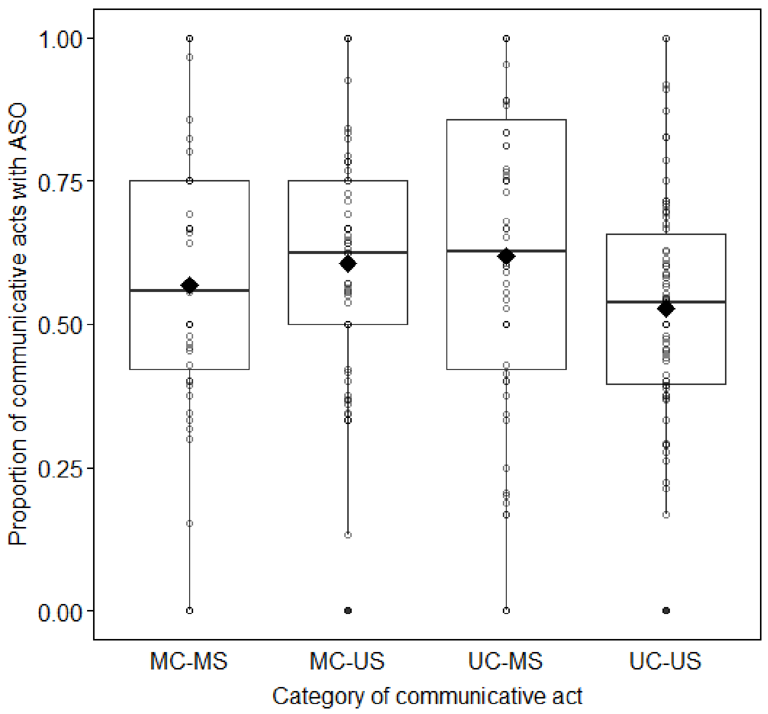

Fig. S4 Proportion of communicative acts receiving an apparently satisfactory outcome (ASO) as a function of communicative act category. Indicated are individual means (circles), population means (filled diamonds), medians (horizontal lines), quartiles (boxes), percentiles (2.5% and 97.5%, vertical lines) and outliers (filled dots).

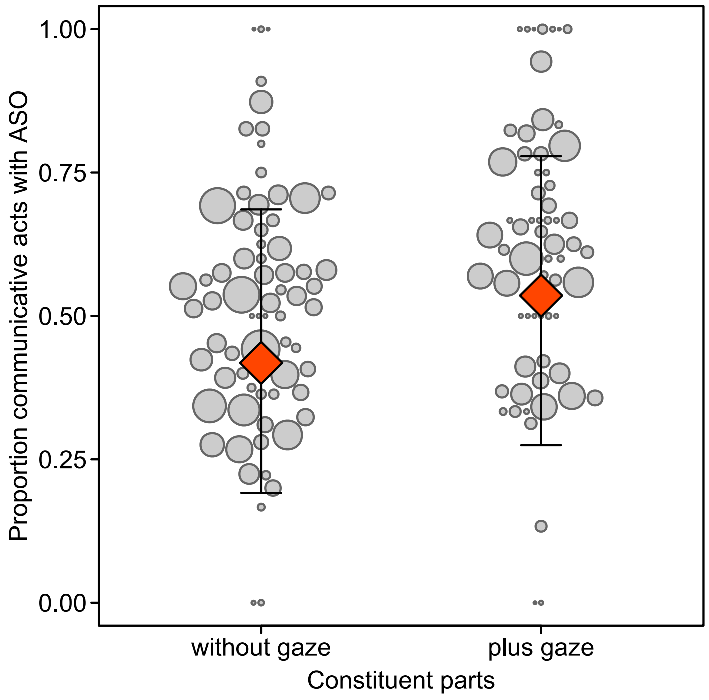

Fig. S5 Proportion of unisensory communicative acts receiving an apparently satisfactory outcome (ASO) as a function of multicomponent use of communicative acts (without/plus recipient-directed gaze). Circles indicate individual means, with circle area representing sample size (range = 1–181). Red diamonds depict model estimates with 95% confidence intervals.

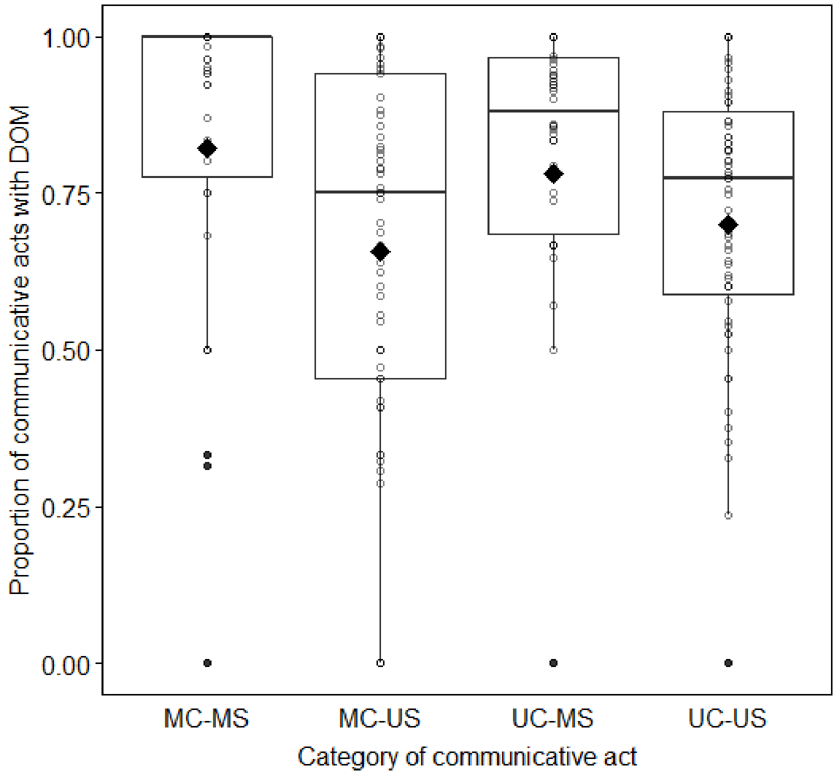

Fig. S6 Proportion of communicative acts whose presumed goal matched the dominant outcome (DOM) as a function of communicative act category. Indicated are individual means (circles), population means (filled diamonds), medians (horizontal lines), quartiles (boxes), percentiles (2.5% and 97.5%, vertical lines) and outliers (filled dots).
