# Supplementary material for: Multicomponent and multisensory communicative acts in orang-utans may serve different functions
Source: Commun Biol. 2021 Jul 27;4:917. doi: 10.1038/s42003-021-02429-y (PMC8316500; doi:10.1038/s42003-021-02429-y)
Supplement: Supplementary file 5 — Reporting Summary [file 42003_2021_2429_MOESM5_ESM.pdf]

nature research

Corresponding author(s): Marlen Fröhlich

Last updated by author(s): Jun 5, 2021

# Reporting Summary

Nature Research wishes to improve the reproducibility of the work that we publish. This form provides structure for consistency and transparency in reporting. For further information on Nature Research policies, see our Editorial Policies and the Editorial Policy Checklist.

## Statistics

For all statistical analyses, confirm that the following items are present in the figure legend, table legend, main text, or Methods section.

| n/a | Confirmed | |
|---|---|---|
| ☐ | ☒ | The exact sample size ($n$) for each experimental group/condition, given as a discrete number and unit of measurement |
| ☐ | ☒ | A statement on whether measurements were taken from distinct samples or whether the same sample was measured repeatedly |
| ☐ | ☒ | The statistical test(s) used AND whether they are one- or two-sided<br>*Only common tests should be described solely by name; describe more complex techniques in the Methods section.* |
| ☐ | ☒ | A description of all covariates tested |
| ☐ | ☒ | A description of any assumptions or corrections, such as tests of normality and adjustment for multiple comparisons |
| ☐ | ☒ | A full description of the statistical parameters including central tendency (e.g. means) or other basic estimates (e.g. regression coefficient) AND variation (e.g. standard deviation) or associated estimates of uncertainty (e.g. confidence intervals) |
| ☐ | ☒ | For null hypothesis testing, the test statistic (e.g. $F$, $t$, $r$) with confidence intervals, effect sizes, degrees of freedom and $P$ value noted<br>*Give P values as exact values whenever suitable.* |
| ☒ | ☐ | For Bayesian analysis, information on the choice of priors and Markov chain Monte Carlo settings |
| ☒ | ☐ | For hierarchical and complex designs, identification of the appropriate level for tests and full reporting of outcomes |
| ☒ | ☐ | Estimates of effect sizes (e.g. Cohen's $d$, Pearson's $r$), indicating how they were calculated |

*Our web collection on statistics for biologists contains articles on many of the points above.*

## Software and code

Policy information about availability of computer code

| | |
|---|---|
| Data collection | Scan sampling was performed with CyberTracker software (v3.486). |
| Data analysis | Video coding was performed using BORIS software (v7.0.4). Statistical analyses were performed using R software (v3.4.1, R Core team) and the following packages: arm (v1.1-25), car (v3.0-10), lsmeans (v2.30-0). |

For manuscripts utilizing custom algorithms or software that are central to the research but not yet described in published literature, software must be made available to editors and reviewers. We strongly encourage code deposition in a community repository (e.g. GitHub). See the Nature Research guidelines for submitting code & software for further information.

## Data

Policy information about availability of data

All manuscripts must include a data availability statement. This statement should provide the following information, where applicable:

- Accession codes, unique identifiers, or web links for publicly available datasets
- A list of figures that have associated raw data
- A description of any restrictions on data availability

The datasets and R code supporting this article are available on GitHub: https://doi.org/10.5281/zenodo.4882719

# Field-specific reporting

Please select the one below that is the best fit for your research. If you are not sure, read the appropriate sections before making your selection.

☐ Life sciences ☐ Behavioural & social sciences ☒ Ecological, evolutionary & environmental sciences

For a reference copy of the document with all sections, see nature.com/documents/nr-reporting-summary-flat.pdf

# Ecological, evolutionary & environmental sciences study design

All studies must disclose on these points even when the disclosure is negative.

| | |
|---|---|
| Study description | To gain insight into the use and function of multi-component communicative acts in great apes, we conducted an observational, quantitative study of intra-specific interactions in wild and captive orang-utans of two different species (Pongo abelii/pygmaeus). We collected video footage and scan samples, which were subsequently analysed with a predefined coding scheme. |
| Research sample | Data were collected at two field sites and five captive facilities (zoos). We observed wild orang-utans at the long-term research sites of Suaq Balimbing and Tuanan (see more information on field sites below), on a population of wild Sumatran (Pongo abelii) and Bornean orang-utans (Pongo pygmaeus wurmbii), respectively. Captive Bornean orang-utans were observed at the zoos of Cologne and Munster, and at Apenheul (Apeldoorn), while Sumatran orang-utans were observed at the zoo of Zurich and at Hellabrunn (Munich) (see EEP studbook for details on captive groups). While captive Sumatran orang-utans were housed in groups of nine individuals each, captive Bornean groups were generally smaller and sometimes included only a mother and her dependent and independent offspring (e.g. Apenheul). Signallers included in this study consisted of 33 Bornean (21 wild/12 captive) and 38 Sumatran orang-utans (20 wild/18 captive; see Tab. S1 for detailed information on subjects and group compositions). Recipients consisted of 81 individuals comprising all age-sex classes. |
| Sampling strategy | Two different behavioural sampling methods were combined: First, intra-specific communicative interactions of all social interactions of the focal as signaller and as receiver with all partners, but also among other conspecifics present were recorded using a digital High-Definition camera (Panasonic HC-VXF 999 or Canon Legria HF M41) with an external directional microphone (Sennheiser MKE600 or ME66/K6). In captive settings with glass barriers, we also used a Zoom H1 Audio recorder that was placed in background areas of the enclosure whenever possible, to enable the recording of auditory communicative acts. Second, using instantaneous scan sampling at ten-minute intervals, we recorded complementary data on the activity of the focal individual, the distance and identity of all association partners, in case of social interactions the interaction partner as well as several other parameters. |
| Data collection | Behavioural data were collected following an established protocol for orang-utan data collection (https://www.aim.uzh.ch/de/orangutannetwork/sfm.html), using focal scan sampling. All observers (MF, NB, CF, CW) were trained to use this protocol and inter-observer reliability tests were conducted after each training phase. MF collected data at both study sites ensuring the use of the same criteria during training. |
| Timing and spatial scale | Focal observations were conducted between November 2017 and October 2018 (Suaq Balimbing: November 2017 – October 2018; Tuanan: January 2018 – July 2018, European zoos: January 2018 – June 2018). At the two field sites, they consisted of full (nest-to-nest) or partial follows (e.g. nest-to-lost or found-to-nest) of mother-infant units, whereas in zoos 6-hour focal follows were conducted. |
| Data exclusions | No data were excluded from the analyses. |
| Reproducibility | No experiments were carried out in this study. |
| Randomization | Not applicable to this study, since it was purely observational. |
| Blinding | All analyses were performed blind in respect to the outcome. |

Did the study involve field work? ☒ Yes ☐ No

## Field work, collection and transport

| | |
|---|---|
| Field conditions | Both field sites consist mainly of peat swamp forest and show high orang-utan densities, with 7 individuals per km2 at Suaq and 4 at Tuanan. |
| Location | We observed wild orang-utans at the long-term research sites of Suaq Balimbing (03°02′N; 97°25′E, Gunung Leuser National Park, South Aceh, Indonesia) and Tuanan (02°15′S; 114°44′E, Mawas Reserve, Central Kalimantan, Indonesia). Captive Bornean orang-utans were observed at the zoos of Cologne and Munster, and at Apenheul (Apeldoorn), while Sumatran orang-utans were observed at the zoo of Zurich and at Hellabrunn (Munich). |
| Access & import/export | The following institutions granted permission to conduct research on wild orang-utans: the Indonesian State Ministry for Research and Technology (RISTEK, 398/SIP/FRP/E5/Dit.KI/XI/2017), the Indonesian Institute of Science (LIPI), the Directorate General of Natural Resources and Ecosystem Conservation – Ministry of Environment & Forestry of Indonesia (KSDAE-KLHK, SI.70/SET/HKST/Kum.I/II/2017), the Ministry of Internal affairs, the Nature Conservation Agency of Central Kalimantan (BKSDA), the local governments in Central Kalimantan, the Kapuas Protection Forest Management Unit (KPHL), the Bornean Orang-utan Survival Foundation (BOSF) and MAWAS in Palangkaraya. |

| | |
|---|---|
| Disturbance | All methods used in this study were non-invasive, with behavioural observations conducted from a minimum distance of 10 m. The research complied with the principles of "Ethical Treatment of Non-Human Primates" as stated by the American Society of Primatologists. |

# Reporting for specific materials, systems and methods

We require information from authors about some types of materials, experimental systems and methods used in many studies. Here, indicate whether each material, system or method listed is relevant to your study. If you are not sure if a list item applies to your research, read the appropriate section before selecting a response.

## Materials & experimental systems

| n/a | Involved in the study | |
|---|---|---|
| ☒ | ☐ | Antibodies |
| ☒ | ☐ | Eukaryotic cell lines |
| ☒ | ☐ | Palaeontology and archaeology |
| ☐ | ☒ | Animals and other organisms |
| ☒ | ☐ | Human research participants |
| ☒ | ☐ | Clinical data |
| ☒ | ☐ | Dual use research of concern |

## Methods

| n/a | Involved in the study | |
|---|---|---|
| ☒ | ☐ | ChIP-seq |
| ☒ | ☐ | Flow cytometry |
| ☒ | ☐ | MRI-based neuroimaging |

## Animals and other organisms

Policy information about studies involving animals; ARRIVE guidelines recommended for reporting animal research

| | |
|---|---|
| Laboratory animals | The study did not involve laboratory animals. |
| Wild animals | We observed wild orang-utans at the long-term research sites of Suaq Balimbing and Tuanan, on a population of wild Sumatran (Pongo abelii) and Bornean orang-utans (Pongo pygmaeus wurmbii), respectively. Captive Bornean orang-utans were observed at the zoos of Cologne and Munster, and at Apenheul (Apeldoorn), while Sumatran orang-utans were observed at the zoo of Zurich and at Hellabrunn (Munich). The study involved subjects of both sexes and of all age classes. |
| Field-collected samples | The study did not involve samples collected from the field. |
| Ethics oversight | The following institutions granted permission to conduct research on wild orang-utans: the Indonesian State Ministry for Research and Technology (RISTEK, 398/SIP/FRP/E5/Dit.KI/XI/2017), the Indonesian Institute of Science (LIPI), the Directorate General of Natural Resources and Ecosystem Conservation – Ministry of Environment & Forestry of Indonesia (KSDAE-KLHK, SI.70/SET/HKST/Kum.I/II/2017), the Ministry of Internal affairs, the Nature Conservation Agency of Central Kalimantan (BKSDA), the local governments in Central Kalimantan, the Kapuas Protection Forest Management Unit (KPHL), the Bornean Orang-utan Survival Foundation (BOSF) and MAWAS in Palangkaraya. |

Note that full information on the approval of the study protocol must also be provided in the manuscript.
