# Supplementary material for: Multicomponent and multisensory communicative acts in orang-utans may serve different functions
Source: Commun Biol. 2021 Jul 27;4:917. doi: 10.1038/s42003-021-02429-y (PMC8316500; doi:10.1038/s42003-021-02429-y)
Supplement: Supplementary file 6 — Description of Supplementary Files [file 42003_2021_2429_MOESM6_ESM.pdf]

Description of Additional Supplementary Files

File name: Supplementary Data S1

Description: Dominant outcomes of communicative acts in relation to research setting and orang-utan species.

File name: Supplementary Data S2

Description: Results of model stability checks.
